# The Number of Metastatic Lymph Nodes is a Useful Predictive Factor for Recurrence after Surgery for Nonmetastatic Nonfunctional Neuroendocrine Neoplasm of the Pancreas

**DOI:** 10.1155/2019/6856329

**Published:** 2019-04-21

**Authors:** G. Capretti, G. Nappo, V. Smiroldo, M. Cereda, B. Branciforte, P. Spaggiari, S. Carrara, P. Preatoni, F. Gavazzi, C. Ridolfi, G. Donisi, A. Lania, A. Zerbi

**Affiliations:** ^1^Pancreatic Surgery Unit, Department of General Surgery, Humanitas Research Hospital, Rozzano, Milan, Italy; ^2^Humanitas Clinical and Research Center-IRCCS, via Manzoni 56, 20089 Rozzano MI, Italy; ^3^Medical Oncology and Hematology Unit, Humanitas Cancer Center, Humanitas Research Hospital, Rozzano, Milan, Italy; ^4^Department of Pathology, Humanitas Research Hospital, Rozzano, Milan, Italy; ^5^Digestive Endoscopy Unit, Division of Gastroenterology, Humanitas Research Hospital, Rozzano, Milan, Italy; ^6^Gastroenterology, Division of Gastroenterology, Humanitas Research Hospital, Rozzano, Milan, Italy; ^7^Endocrinology Unit, Humanitas Research Hospital, Rozzano, Milan, Italy; ^8^Department of Biomedical Sciences, Humanitas University, Milan, Italy

## Abstract

Nodal involvement (actually categorized as positive or negative) is an important prognostic factor after surgery for pancreatic neuroendocrine neoplasms (pNENs). We aim to evaluate the predictive role of the number of nodal metastases after pancreatic resection for pNENs. We analyzed from a prospectively maintained database all pancreatic resections for nonmetastatic nonfunctioning pNENs performed in our institution from 2011 to 2016. According to the number of nodal metastases, enhancing the actual categorization, we distinguished the following: N0, no nodal metastases; N1, 1-3 metastatic lymph nodes; and N2, metastases in 4 or more regional lymph nodes. Recurrence and disease-free survival (DFS) were evaluated. The predictive value in terms of recurrence for each clinicopathological data, including the number of metastatic lymph nodes, was calculated. Univariate and multivariate analyses were conducted. 77 patients underwent pancreatic surgery for pNENs. N0, N1, and N2 resections were found in 52 (67.5%), 16 (20.8%), and 9 (11.7%) cases, respectively. Mean follow-up of the entire cohort was 48 (±25) months. The recurrence rate was 11.8%, and the mean time of recurrence was 12 (±14) months. DFS was 83.7 months (76.0 - 91.5). At a univariate analysis, factors associated with recurrence were mitotic count (OR 1.19, *p* = 0.001), Ki67 value (OR 1.06, *p* = 0.001), the presence of nodal metastases (OR 11.54, *p* = 0.002), and metastases in 4 or more regional lymph nodes (N2) (OR 30.19, *p* = 0.002). At a multivariate analysis, only mitotic count (OR 1.51, *p* = 0.005) and N2 resection (OR 134.74, *p* = 0.002) were found to be predictive factors of recurrence. The number of metastatic lymph nodes and mitotic count is the most significant predictive factors of recurrence after pancreatic surgery for nonmetastatic nonfunctioning pNENs.

## 1. Introduction

Pancreatic neuroendocrine neoplasms (pNENs) are rare neoplastic diseases, with an estimated incidence of 1/100000 people and account for about 1-2% of all pancreatic tumors [1]. Radical surgery represents the first-line potentially curative treatment for locoregional pNENs [2]. During the last years, several pathological factors, including tumor size, tumor grading, and proliferation index (ki-67), have been identified as prognostic factors after resection for nonmetastatic pNENs (pNENs without distant metastases) [3].

The presence of nodal metastases is currently considered as one of the most powerful prognostic factors [4], and an adequate lymphadenectomy is recommended for pNENs > 2 cm and G3 forms [5]. Recently, some studies demonstrated that not only nodal involvement but also the number of metastatic lymph nodes is a significant prognostic factor after the resection of locoregional pNENs [6, 7]. Adopting the latest UICC/AJCC TNM classifications of pancreatic ductal adenocarcinoma (PDAC) [8, 9] that differentiates N1 and N2 resections according to the number of metastatic lymph nodes (N1: 1-3 positive lymph nodes; N2: metastases in 4 or more regional lymph nodes), these studies demonstrated that patients with more than 3 metastatic nodes had the significant worse survival [6, 7].

The aim of the current study was to evaluate the prediction role of nodal involvement and of the number of metastatic lymph nodes in our series of standard pancreatic resections for sporadic nonmetastatic pNENs.

## 2. Materials and Methods

All consecutive standard pancreatic resections performed with curative intent for nonmetastatic nonfunctioning pNENs between 2011 and 2016 at our center were retrospectively analyzed from a prospective collected database. Functioning metastatic pNENs and those associated with genetic syndrome were excluded.

All pancreatic resections were performed by expert pancreatic surgeons in a high-volume pancreatic center. Standard resections included pancreaticoduodenectomy (PD), distal pancreatectomy (DP), and total pancreatectomy (TP). The different resections were performed according to the preoperative work-up and to the decision of multidisciplinary board. All PD and TP were performed with an open approach, while a laparoscopic approach was adopted for selected cases of DP. Standard lymphadenectomy was performed in all resections [10]. In all PDs, a pancreaticojejunal anastomosis was performed.

Database was queried for demographic data, perioperative, intraoperative, and postoperative detail findings. Postoperative complications were collected and defined according to Clavien-Dindo's classification [11] with grade III or higher considered as major. Postoperative Pancreatic Fistula (POPF) was classified according the ISGPS classification [12].

Pathological reports were collected and evaluated. T and N stages were classified according to the current ENETS classification [13]. The number of examined and positive lymph nodes was retrieved from histological reports. Lymph node ratio (LNR), defined as the ratio between positive and examined lymph nodes, was also calculated. Moreover, adopting the UICC and AJCC classifications for pancreatic ductal adenocarcinoma [8, 9], according to the number of positive lymph nodes, we distinguished two different subclasses of N resections: N1, 1-3 metastatic lymph nodes; and N2 metastases in 4 or more regional lymph nodes. Immunostaining routinely included synaptophysin, chromogranin, and Ki67 proliferative index assessed in all the surgical specimens by MIB1 antibody staining and expressed as the percentage of cells with positive nuclear staining in 2000 cells counted in the area of the highest nuclear labelling. Tumor grade was reclassified by pathologist according to the pancreas WHO Classification of Tumours of Endocrine Organs, 4th edition 2017 [14].

According to that, pNEN G1 has a mitotic count < 2/10 high power fields (HPFs) and Ki-67 index < 3%, pNEN G2 has a mitotic count 2–20/10 HPFs and/or Ki-67 index 3–20%, pNEN G3 has a mitotic count > 20/10 HPFs and/or a Ki-67 index > 20% and is classified as well differentiated by pathologist, and pNEC has a mitotic count > 20/10 HPFs and/or a Ki-67 index > 20% but is classified as poorly differentiated.

Mitotic counts was assessed in at least 50 HPFs in most mitotically active areas, and Ki-67 labeling index was measured in 500-2000 cells in the most proliferative “hot spot” areas.

Taking in consideration the discrepancies between mitotic count and Ki-67, the final grade was based on which ever index places the tumor in the highest grade category.

R1 resection was defined as the presence of neoplastic involvement of the evaluated margin (0 mm rule).

All cases were discussed at a multidisciplinary board for the decision of eventual treatments and of timing of follow-up. Generally, a 6-month follow-up including at least a high-quality imaging procedure was proposed to all the patients for the first 2 years and yearly thereafter as a minimum for at least 5 years from surgery. Ga68-PET was usually repeated at one year from surgery in neoplasm positive to the preoperative evaluation.

Information regarding general health status, disease recurrence, or progression was collected during scheduled outpatient visits. Disease-free survival (DFS) was defined as the time from diagnosis to first evidence of disease recurrence at imaging and was censored at the last follow-up date if no events had occurred. Overall survival (OS) was defined as the time from diagnosis to the last follow-up.

### 2.1. Statistical Analysis

Data were analyzed using the IBM SPSS software version 13 and are expressed as percentage or mean ± standard deviation. Descriptive and inferential statistics were carried out with the parametrical analytical models adequate for the type of variable studied (e.g., *T*-student, chi-square). DFS was assessed using the Cox regression model and Kaplan-Meier curves with log-rank comparison. Odds ratio (OR) was reported with the 95% confidence interval (CI). Univariate and multivariate analyses were conducted. Statistical significance was stated with a *p* value < 0.05. No analysis of OS was carried out due to a single disease-specific event during the follow-up.

## 3. Results

During the study period, 77 pancreatic resections were performed for nonmetastatic nonfunctioning pNENs (36 pNETs G1, 37 G2, 3 G3, and 1 pNEC G3) and were included in the analysis: 22 PD (28.6%), 50 DP (64.9%), and 5 TP (6.5%).

Demographic and clinicopathological features are listed in Table 1. Mean Ki67 and mitotic count were 6.1 ± 11.9% and 2.5 ± 5, respectively. The mean number of harvested lymph nodes was 12.6 ± 10; according to the type of resection, the mean harvested lymph nodes were 21.0 ± 9, 8.1 ± 7.9, and 19.8 ± 14 in PD, DP, and TP, respectively (*p* < 0.001). The overall rate of nodal metastases was 32.5%. According to the number of metastatic lymph nodes, resections were classified as N1 and N2 in 16 (20.8%) and 9 (11.7%) cases, respectively. Mean LNR was 0.09 ± 0.2, without a significant difference between the surgical procedures.

Only in one case 90-day postoperative mortality was recorded (1.3%): the patient died in postoperative day 7 after TP for a septic shock. Major postoperative complications occurred in 15 cases (19.5%); the POPF rate was 22.2% (31.8% and 18.0% in PD and DP, respectively (*p* < 0.001)). The mean postoperative length of stay was 10 days ± 5, and the readmission rate was 7.8%.

The mean follow-up of the entire cohort was 40.8 ± 25 months. During the follow-up period, only 1 patient died due to disease's progression five months after the surgical procedure: it was the case of an aggressive pNEN with a value of Ki67 of 90%, an outlier value if compared with the entire cohort (surgical indication was given due to the response rate of the disease to previous systemic therapies). Three other patients died during the follow-up period due to other causes not related to disease. The recurrence rate was 11.7% (8 cases), and the mean time of recurrence was 12 ± 14 months. DFS was 83.7 months (76.0 - 91.5).

The univariate analysis of DFS predictive factors was described in Table 2. At a univariate analysis, Ki67 values (OR 1.06 (1.03-1.10)), mitotic count values (OR 6.28 (2.01-19.57)), and lymph node metastases (OR 11.54 (1.39-95.95) were identified as significant prognostic factors (*p* < 0.05). When we considered the number of nodal metastases, at a univariate analysis, N2 resection was a significant prognostic factor (OR 30.12 (2/44-244.94), *p* < 0.002). No significant correlation was found with the patients' characteristics or type of procedure.

**Figure 1:**
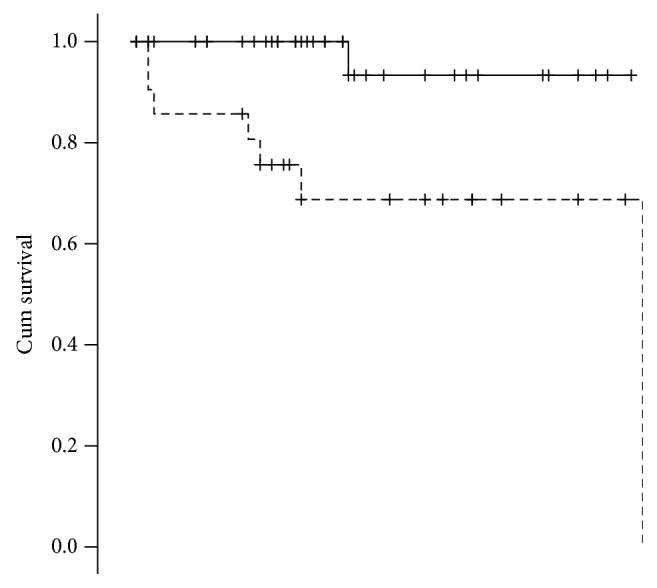
DFS Kaplan-Meier for lymph node involvement yes/no. *p* = 0.004; mean survival, no: 81.800 (75.741-87.859); yes: 64.404 (47.923-80.886).

**Figure 2:**
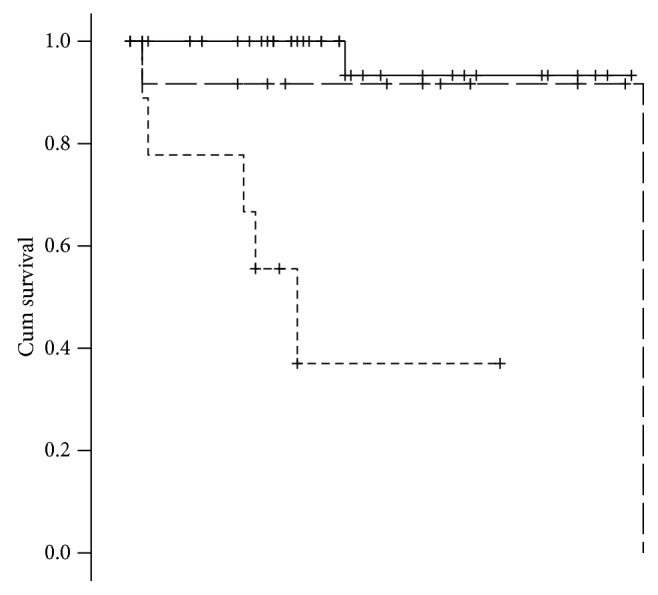
DFS Kaplan-Meier for lymph node involvement proposed categorization. *p* < 0.001; mean survival, N0: 81.800 (75.741-87.859); N1: 80.000 (61.423-98.577); N2: 34.148 (17.417-50.879).

**Figure 3:**
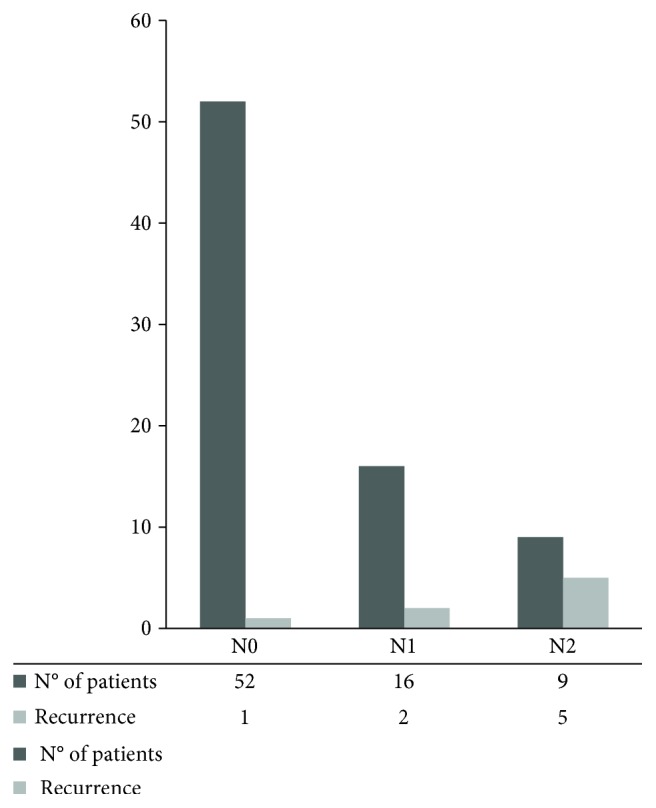
Disease recurrences between N groups.

The better ability to catch variation in DFS of the proposed nodal involvement categorization is also clear evaluating the Kaplan-Meier for DFS of this different grouping variables (Figures 1 and 2), and the mere distribution or relapse among the groups (Figure 3).

In a model accounting for the mitotic count, the Ki67 value, and the presence of lymph node metastases, at a multivariate analysis, only mitotic count remains an independent prognostic factor (OR 1.51 (1.0-2.29)), while the presence of metastatic lymph nodes loses its significance (OR 26.71 (0.60-1180.48)). Performing the multivariate analysis adopting LNR instead of the presence of nodal metastases, LNR seems a more robust variable but still not significant (OR 11.60 (0.91-148.04)). On the other hand, adopting the proposed categorization (N0, N1, and N2), at the same multivariate analysis, N2 resection was found to be an independent prognostic factor (OR 134.74 (2.19-8261.17). In all these three evaluated models, at a multivariate analysis, mitotic count remains an independent prognostic factor.

## 4. Discussion

The correct prognostic evaluation of a neoplastic patient is crucial to determine his specific cure pathway. This aspect is even more relevant in patients affected by pNENs: in fact, these patients could have a very long survival [3], but they could also require major surgical procedures (with a nonnegligible postoperative morbidity and mortality rate, especially in low-volume centers [15]) Results of our study confirmed that standard pancreatic resections for pNENs are challenging procedures even in a referral center: in our series, we reported a postoperative major morbidity of 19.5% and a mortality rate of 1.3%.

Many different classifications for pNENs have been proposed, with the aim to frame in the best way the patients' prognosis [16]. Nodal involvement is currently recognized as one of the most important prognostic factors [5]. During the last years, the prognostic impact of the number of metastatic lymph nodes has been demonstrated for PDAC [17]; according to these results, 8th editions of the AJCC and UICC TNM staging systems were recently modified [8, 9], distinguishing N1 and N2 resections according to the number of nodal metastases (N1: 1-3 nodal metastases; N2: metastases in 4 or more regional lymph nodes). Recently, this aspect was translated in the field of pNENs: some studies reported that not only the presence but also the number of nodal metastases seems to be an independent prognostic factor after resection for pNENs [6, 7]. Partelli et al. [6], in a retrospective study evaluating patients undergoing PD for pNENs, demonstrated that N0 resections had a 3-year DFS rate of 89% compared with 83% and 75% in N1 and N2 resections, respectively (*p* < 0.001). Similar results were found by Zhang et al. [7] evaluating a national retrospective database (SEER) number of metastatic lymph nodes seemed more meaningful than the lymph node metastases status as prognostic factor for DFS (≤3 vs. >3 nodal metastases: 104.829 ± 1.455 months vs. 85.443 ± 3.938 months, respectively). Our study confirmed these results, demonstrating that a number of nodal metastases >3 is able to better discriminate the patients' prognosis: mean DFS was 80.0 (75.74-87.85) and 34.15 (17.42-50.88) months for N1 and N2 patients, respectively (*p* < 0.001). At a multivariate analysis, mitotic count (OR 1.51, *p* = 0.005) and number of nodal metastases (OR 134.74, *p* = 0.002) were found to be the only independent prognostic factors. Mitotic count is theoretically a less reproducible variable with respect to Ki67 proliferation index, despite that, it perform better in the multivariate model. Some aspect of collinearity and the low recurrence rate should be taken into account when evaluating this result.

Interestingly, in our series N0 and N1 patients showed similar mean DFS (81.80 (75.74-87.86) and 80.0 (61.42-98.58) months, respectively). This result demonstrated that N1 patients have a biological behaviour more similar to N0 patients, if compared with N2 ones. Evaluating together N1 and N2 patients, we could risk not only an overall prognostic ability but also to neglect a clinical relevant subgroup of patients that could benefit of a different management.

This study has several limitations. The first one is its retrospective nature. Another weak points are the low number of recurrence in our series that could weaken the statistical power of the study. Unfortunately, national database usually lacks the specific data of number of metastatic lymph nodes to answer this query. As shown in the national retrospective analysis performed by Zhang et al. [7], more than half of the patients were excluded for the lack of this data. In order to validate our results, a large multicentric study is needed.

## 5. Conclusions

The number of metastatic lymph nodes in nonfunctioning resected pNENs seem to be a better prognostic factor, if compared with the simple presence of nodal metastases. This finding should be taken into account for the development of the actual staging classifications.

## Figures and Tables

**Table 1:** Characteristics of the patients and histopathological features of the neoplasms.

Patients	77
Gender (M/F)	45/32
Mean age (yrs)	56.8 ± 15
BMI	25.2 ± 3
ASA score	
1-2	66 (85.7%)
3-4	9 (14.3%)
Type of surgery	
PD	22 (28.6%)
DP	50 (64.9%)
TP	5 (6.5%)
Tumor	
T1–T2	46 (59.7%)
T3–T4	31 (40.3%)
Resection margin	
R0	72 (93.5%)
R1	5 (6.5%)
Grading	
NET G1	36 (46.8%)
NET G2	37 (48.1%)
NET G3	3 (3.9%)
NEC G3	1 (1.2%)
Mitotic count	
<2	54 (70.1%)
2-20	21 (27.3%)
>20	2 (2.6%)
Ki67	
<3%	36 (46.8%)
3-20%	37 (48.1%)
>20%	4 (5.1%)
Number of harvested lymph nodes (mean ± SD)	12.6 ± 10
Nodal involvement	
N0	52 (67.5%)
N1	16 (20.8%)
N2	9 (11.7%)
LNR (mean ± SD)	0.09 ± 0.2

PD: pancreaticoduodenectomy; DP: distal pancreatectomy; TP: total pancreatectomy. Continuous data are expressed as mean ± standard deviation; nodal invasion is reported as proposed in the methods section. pNENs: pancreatic neuroendocrine neoplasms; pNECs: pancreatic neuroendocrine carcinomas.

**Table 2:** Univariate analysis of DFS predictive factors.

	Univariate *p* value	OR (95% CI)
T1-T2 T3-T4	0.193	80.88 (0.108-60744.255)
Mitotic count	0.001	1.193 (1.079-1.321)
R	0.460	2.225 (0.266-18.602)
Ki 67	0.001	1.062 (1.027-1.099)
Positive nodal status	0.024	11.543 (1.389-95.950)
Nodal status		
N0	Reference	Reference
N1	0.473	2.771 (0.172-44.736)
N2	0.002	30.191 (3.440-264.938)
Number of harvested lymph nodes	0.077	1.053 (0.994-1.116)
Lymph nodes ratio	0.004	14.017 (2.307-85.160)
Microvascular invasion	0.057	7.748 (0.942-63.723)
Perineural invasion	0.068	5.315 (0.886-31.891)
Necrosis	0.096	4.298 (0.772-23.921)

## Data Availability

The data used to support the findings of this study are available from the corresponding author upon request.

## References

[1] Milan S. A., Yeo C. J. (2012). Neuroendocrine tumors of the pancreas. *Current Opinion in Oncology*.

[2] Falconi M., Eriksson B., Kaltsas G. (2016). ENETS consensus guidelines update for the management of patients with functional pancreatic neuroendocrine tumors and non-functional pancreatic neuroendocrine tumors. *Neuroendocrinology*.

[3] Genç C. G., Jilesen A. P., Partelli S. (2018). A new scoring system to predict recurrent disease in grade 1 and 2 nonfunctional pancreatic neuroendocrine tumors. *Annals of Surgery*.

[4] Curran T., Pockaj B. A., Gray R. J., Halfdanarson T. R., Wasif N. (2015). Importance of lymph node involvement in pancreatic neuroendocrine tumors: impact on survival and implications for surgical resection. *Journal of Gastrointestinal Surgery*.

[5] Hashim Y. M., Trinkaus K. M., Linehan D. C. (2014). Regional lymphadenectomy is indicated in the surgical treatment of pancreatic neuroendocrine tumors (PNETs). *Annals of Surgery*.

[6] Partelli S., Javed A. A., Andreasi V. (2018). The number of positive nodes accurately predicts recurrence after pancreaticoduodenectomy for nonfunctioning neuroendocrine neoplasms. *European Journal of Surgical Oncology*.

[7] Zhang X., Lu L., Shang Y. (2017). The number of positive lymph node is a better predictor of survival than the lymph node metastasis status for pancreatic neuroendocrine neoplasms: a retrospective cohort study. *International Journal of Surgery*.

[8] Amin M. B., Edge S., Greene F. (2016). *AJCC Cancer Staging Manual*.

[9] Brierley J. D., Gospodarowicz M. K., Wittekind C. (2017). *TNM Classification of Malignant Tumours*.

[10] Tol J. A., Gouma D. J., Bassi C. (2014). Definition of a standard lymphadenectomy in surgery for pancreatic ductal adenocarcinoma: a consensus statement by the International Study Group on Pancreatic Surgery (ISGPS). *Surgery*.

[11] Dindo D., Demartines N., Clavien P. A. (2004). Classification of surgical complications: a new proposal with evaluation in a cohort of 6336 patients and results of a survey. *Annals of Surgery*.

[12] Bassi C., Marchegiani G., Dervenis C. (2017). The 2016 update of the International Study Group (ISGPS) definition and grading of postoperative pancreatic fistula: 11 years after. *Surgery*.

[13] Rindi G., Klöppel G., Alhman H. (2006). TNM staging of foregut (neuro)endocrine tumors: a consensus proposal including a grading system. *Virchows Archiv*.

[14] Lloyd R. V., Osamura R. Y., Kloppel G., Rosai J. (2017). *WHO Classification of Tumours: Pathology and Genetics of Tumours of Endocrine Organs*.

[15] Balzano G., Zerbi A., Capretti G., Rocchetti S., Capitanio V., di Carlo V. (2008). Effect of hospital volume on outcome of pancreaticoduodenectomy in Italy. *British Journal of Surgery*.

[16] Teo R. Y. A., Teo T. Z., Tai D. W. M., Tan D. M., Ong S., Goh B. K. P. (2019). Systematic review of current prognostication systems for pancreatic neuroendocrine neoplasms. *Surgery*.

[17] Strobel O., Hinz U., Gluth A. (2015). Pancreatic adenocarcinoma: number of positive nodes allows to distinguish several N categories. *Annals of Surgery*.

